# *M6PR*/*IGF2R* double-knockout supplies therapeutic sources for mucolipidosis II and Niemann-Pick C2

**DOI:** 10.1016/j.jbc.2026.113511

**Published:** 2026-08-31

**Authors:** Toshiharu Fujita, Seigo Terawaki, Takahito Moriwaki, Takanobu Otomo

**Affiliations:** Department of Molecular and Genetic Medicine, Kawasaki Medical School, Kurashiki, Okayama, Japan

**Keywords:** enzyme replacement therapy, GlcNAc-1-phosphotransferase, I-cell disease, lysosomal storage disorder, lysosome, mannose 6-phosphate, mucolipidosis II, Niemann-Pick C2

## Abstract

Lysosomal storage disorders (LSDs) are intractable rare diseases caused by lysosomal dysfunction due mainly to defects in lysosomal enzyme genes. For many lysosomal enzymes to be transported correctly into lysosomes, their mannose-6-phosphate (M6P) labeling by GlcNAc-1 phosphotransferase is crucial. M6P-modified lysosomal enzymes are captured by M6P receptors and transported to lysosomes. The M6P-dependent pathway is utilized not only for newly synthesized lysosomal enzymes but also for the intracellular transport of exogenously administered lysosomal enzymes to lysosomes. In this study, we performed gene knockouts targeting the *M6PR* and *IGF2R* genes, which encode the M6P receptors. Cells with a double knockout of these genes secrete M6P-modified proteins into the culture supernatant double KO (dKO sup), and we investigated its potential for therapeutic application. I-cell disease (mucolipidosis II, ML-II) arises from GNPT deficiency and is a disorder in which dozens of lysosomal enzymes are deficient within the cell. Treating ML II cells with the dKO sup restored lysosomal enzyme activity, reduced inclusion bodies, and improved autophagic function. ML II patient-derived fibroblasts exhibited increased lysosomal enzyme activities, normalized morphology, and improved cryo-viability with the treatment. Niemann-Pick disease type C2 (NPC2) protein is not a lysosomal enzyme but functions cooperatively with NPC1 protein to mediate cholesterol transport in lysosomes. NPC2 deficiency causes NPC2, and the dKO sup restored the cellular function of the disease by supplementing the NPC2 protein. These results indicate that M6PR/IGF2R double-deficient cells provide a simple platform for supplying M6P-modified proteins that can be applied to the treatment of a broad range of LSDs.

Lysosomes are small vesicular organelles that play a crucial role in cellular homeostasis by degrading and recycling various biological macromolecules (1, 2). They contain a diverse array of acid hydrolases that degrade proteins, lipids, carbohydrates, or nucleic acids. Mutations in the genes encoding these lysosomal enzymes lead to decreased degradation activities and result in lysosomal storage disorders (LSDs) (3, 4, 5). Enzyme replacement therapy (ERT) is currently established in several LSDs, including Gaucher disease, Fabry disease, Pompe disease, lysosomal acid lipase (LAL) deficiency, and part of neuronal ceroid lipofuscinoses and mucopolysaccharidoses (4, 6). ERT is aimed at restoring cell functions by administering the recombinant enzymes that are genetically deficient in patients as a drug, to promote the breakdown of accumulated substances in the lysosomes.

In addition to genetic abnormalities in hydrolytic enzymes, deficiencies in other lysosomal functional proteins involved in lysosomal enzyme transport, activation, and substrate or metabolite delivery can also cause LSDs. Disruptions of the machinery responsible for transporting enzymes to the lysosome result in the loss of multiple hydrolytic enzymes in the lysosomes. Most of the newly synthesized lysosomal enzymes are modified with mannose 6-phosphate (M6P) to the non-reducing end of the N-linked oligosaccharide by UDP-N-acetylglucosamine--lysosomal-enzyme N-acetylglucosaminephosphotransferase (GlcNAc-1-phosphotransferase, hereafter GNPT [EC 2.7.8.17]) in the Golgi apparatus. The M6P-modified enzymes are captured by the M6P receptors in the trans-Golgi network and are transported to lysosomes *via* clathrin-coated vesicles (7). A functional defect in GNPT causes impaired M6P modification of lysosomal proteins, leading to their disrupted transport to lysosomes and their secretion into the extracellular space.

Mucolipidoses type II and III (ML II, ML III) are caused by a genetic deficiency of GNPT, where undigested substrates accumulate in lysosomes, forming inclusion bodies in the cytosol (8, 9, 10, 11). Niemann-Pick disease type C2 (NPC2) is caused by a genetic defect of the *NPC2* gene, which works for transporting free cholesterol in the lysosome in coordination with NPC1. These diseases are not lysosomal enzyme deficiencies, and fundamental treatment, such as ERT, has not been developed yet. Here, we report that cells depleted of two types of M6P receptors, M6PR and IGF2R, *via* genome editing secrete M6P-modified proteins into the culture supernatant. These secreted proteins not only serve as a source of ERT for ML II, but can also be applicable for the treatment of other enzyme-deficient LSDs and NPC2, by restoring the cellular function of the patients.

## Results

### Depletion of M6P receptors releases lysosomal enzymes into the extracellular space

Components of GNPT are encoded by two genes, *GNPTAB* and *GNPTG*. Biallelic pathogenic variants in either of these genes cause ML II or its attenuated form, mucolipidosis type III (11, 12, 13). GNPT adds GlcNAc-P to the glycan chain of lysosomal enzymes, and the N-acetylglucosamine-1-phosphodiester alpha-N-acetylglucosaminidase (uncovering enzyme, [EC 3.1.4.45]) removes the terminal GlcNAc from the glycan chain, exposing the M6P residue on the lysosomal enzyme (14, 15). The uncovering enzyme is encoded by the *NAGPA* gene, and no diseases caused by mutations in this gene have been reported in humans (14, 16). To understand the interaction between the M6P residue and M6P receptor in lysosomal enzyme transport and to develop therapeutic strategies for ML II and ML III, gene-modified cells were established using CRISPR/Cas9 (17) and analyzed.

In the *GNPTAB* or *GNPTG* gene knocked out (KO) HeLa cells, intracellular lysosomal enzyme activities were significantly reduced for α-mannosidase, α-fucosidase, α-galactosidase, β-hexosaminidases, β-glucuronidase, and α-iduronidase (Fig. S1*A*). As β-glucosidase is known to be transported to lysosomes in an M6P-independent manner, the intracellular enzyme activity in *GNPTAB* or *GNPTG* KO cells was intact in this study (Fig. S1*A*), which is consistent with the previous report (18, 19). The β-galactosidase activity was significantly reduced in *GNPTAB* KO cells but not in *GNPTG* KO cells. The gamma subunit of GNPT is suggested to recognize substrates, while the alpha and beta subunits possess phosphotransferase activity (14, 20). The mechanism by which intracellular β-galactosidase activity is not reduced by *GNPTG* KO remains unclear.

In *NAGPA* KO cells, intracellular activities of β-hexosaminidase measured using the 6-sulfated (6S) substrate and α-iduronidase were reduced, but no apparent decrease was observed for other enzymes (Fig. S1*A*). To date, human disease linked to *NAGPA* gene deficiency has not been reported, and *NAGPA* KO mice grow normally without histologic abnormalities (16). These findings suggest that the *NAGPA* gene had minimal effects on the transport of most lysosomal hydrolases.

To verify the effects of clonal variation on the phenotype, cell lines with four different biallelic pathogenic mutations introduced into the *GNPTAB* gene were established and tested (Table S1); all of them showed similar reduced enzyme activity profiles (Fig. S1*B*) and complete impairment of autophagic function (Fig. S1*C*), consistent with the previous studies (21, 22, 23). These results indicate that the model cells properly mimic patient cells, and thus clone #1 was used for the subsequent investigations in this study.

The M6P residue on the lysosomal enzymes is recognized and captured by the M6P receptors in the trans-Golgi network, initiating the journey to lysosomes. Two types of M6P receptors are known: cation-dependent M6P receptor (encoded by the *M6PR* gene) that requires divalent cations to efficiently recognize lysosomal hydrolases, and cation-independent M6P receptor (insulin-like growth factor II receptor, encoded by the *IGF2R* gene) that can recognize enzymes without requiring cations (24). To investigate the impact of M6P receptor deficiency on lysosomal enzyme transport, we established *M6PR* KO, *IGF2R* KO, and *M6PR*/*IGF2R* double KO (dKO) cells and measured the lysosomal enzyme activities in the culture medium of these cells.

*M6PR* KO had little effect on enzyme activity in the culture medium, whereas *IGF2R* KO represented significant increases in activities in part of the lysosomal enzymes compared with WT cells (Fig. 1*A*). In *M6PR*/*IGF2R* dKO cells, further increases in enzyme activities were observed in the culture medium for almost all enzymes tested (Fig. 1*A*), which are comparable to those of *GNPTAB* KO cells. Conversely, the intracellular enzyme activities other than β-glucosidase of *GNPTAB* KO and *M6PR*/*IGF2R* dKO cells significantly decreased compared with the WT cells (Fig. 1*B*). These results indicated that the various lysosomal enzymes transported in the M6P-dependent manner were secreted into the supernatant of *M6PR*/*IGF2R* dKO cells. To assess the impact of enzyme transport deficiency on lysosomal degradation substrates, we also examined electron micrographs of the cells tested above. The results showed that abnormal lysosomes, with an increase in number and/or size, were observed in the *M6PR/IGF2R* and *GNPTAB* KO cells (Fig. 1*C*).

**Figure 1:**
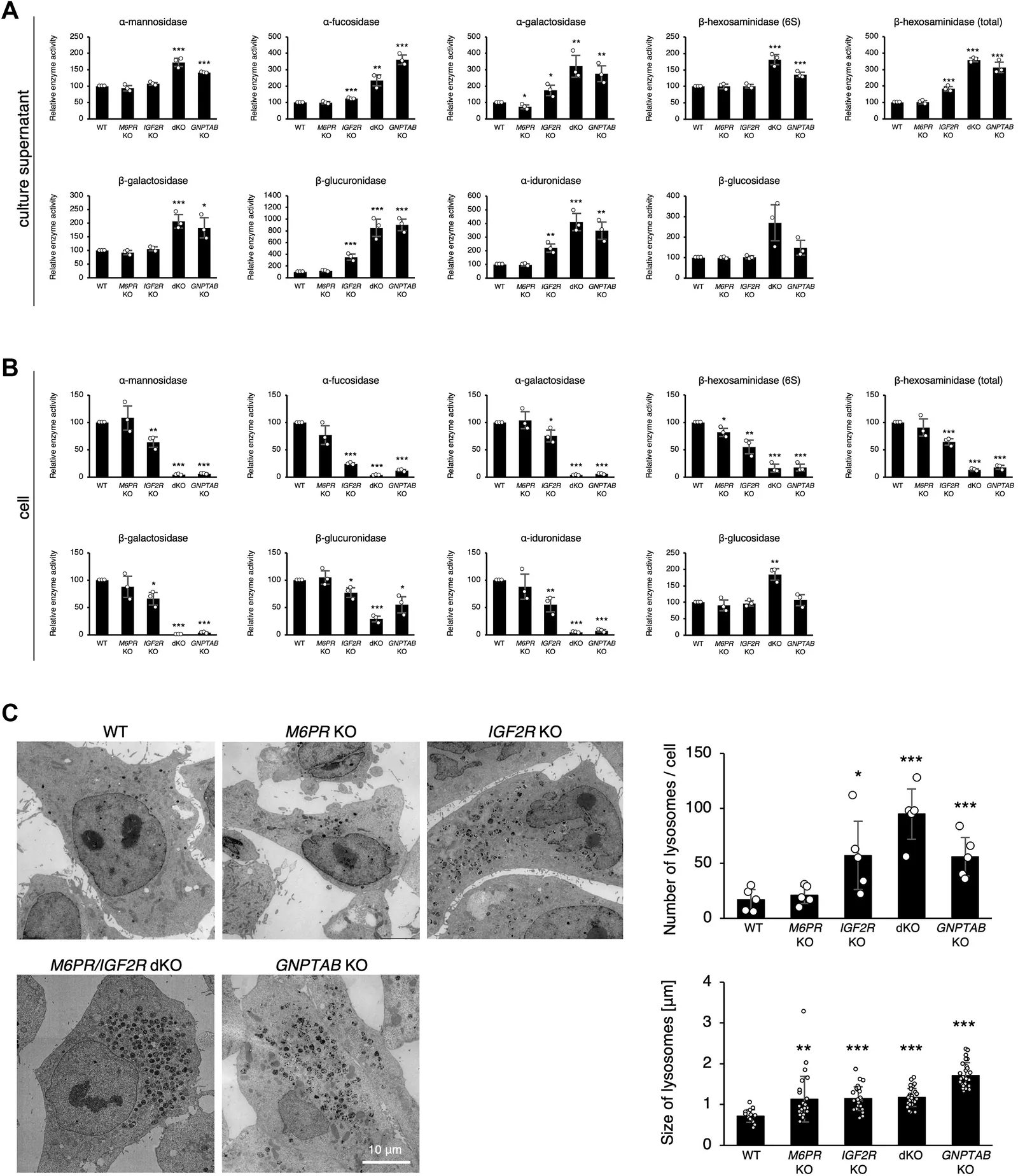
**Impact of M6P receptor depletion on extra- and intracellular lysosomal enzyme activity and morphology.***A*, lysosomal enzyme activities in the culture supernatant of KO cells determined by a fluorometric assay using 4-methylumbelliferyl substrates (assay). *B*, lysosomal enzyme activities in KO cells. Cells and culture supernatants were collected from each dish 4 days after replacement with fresh culture medium. WT, nonedited HeLa cell. The relative activities of each enzyme relative to WT are shown in separate *bar graphs* (mean ± SD, n = 3). The *white circles* represent data points from each independent experiment (*A* and *B*). *C*, electron micrographs of mannose 6-phosphate receptor-knockout cells. Representative images of WT, *M6PR* KO, *IGF2R* KO, *M6PR*/*IGF2R* dKO, and *GNPTAB* KO cells are shown. Lysosomes, identified as electron-dense vesicular structures, were quantified from five independent micrographs per genotype. Lysosomal diameters were measured using Fiji, with at least five lysosomes analyzed per micrograph (≥25 lysosomes per genotype). Data are presented as mean ± SD. The *white circles* represent data points for the number of lysosomes in each cell (*top panel*) and the size of each lysosome (*bottom panel*). Statistical significance was determined by comparing the two groups using Student’s *t* test, with the WT group as the reference. (∗*p* < 0.05, ∗∗*p* < 0.01, ∗∗∗*p* < 0.005).

The results above demonstrate that lysosomal enzyme transport mediated by M6P is impaired when either the M6P modification on the enzymes or the M6P receptor is deficient. Consequently, the enzymes are secreted outside the cell, causing the cells to exhibit a similar phenotype.

### Proteomic characterization revealed the secretion of lysosomal proteins from GNPTAB KO and M6PR/IGF2R dKO cells

All cells secrete various proteins *via* exocytosis and exosome-mediated pathways, and so on. To know the profiles of proteins, which are secreted in the culture supernatant of *M6PR*/*IGF2R* dKO cells (hereafter dKO sup), and to determine their relative abundances, we performed proteomic analysis. Using serum-free medium, we collected culture supernatants from WT, *M6PR*/*IGF2R* single or double KO, and *GNPTAB* KO cells and conducted comprehensive protein profiling by LC-MS/MS (Supplemental data set). The results revealed a marked enrichment of lysosomal proteins in the culture supernatants of *GNPTAB* KO cells and *M6PR*/*IGF2R* dKO cells compared to those of WT cells. A total of 3088 translational products derived from different genes were detected from the samples, and the known lysosomal proteins were highly ranked in abundance (Fig. 2*A*). Among the top 100 detected proteins, 21 were major lysosomal proteins, such as proteases and glycosidases. Furthermore, an additional 11 lysosomal enzymes were identified within the top 300 or so (Fig. 2*B*). The profiles of the cell culture supernatants from *GNPTAB* KO cells and *M6PR*/*IGF2R* dKO cells were very comparable, indicating that disruption of the M6P-dependent pathway similarly leads to the extracellular secretion of lysosomal proteins.

**Figure 2:**
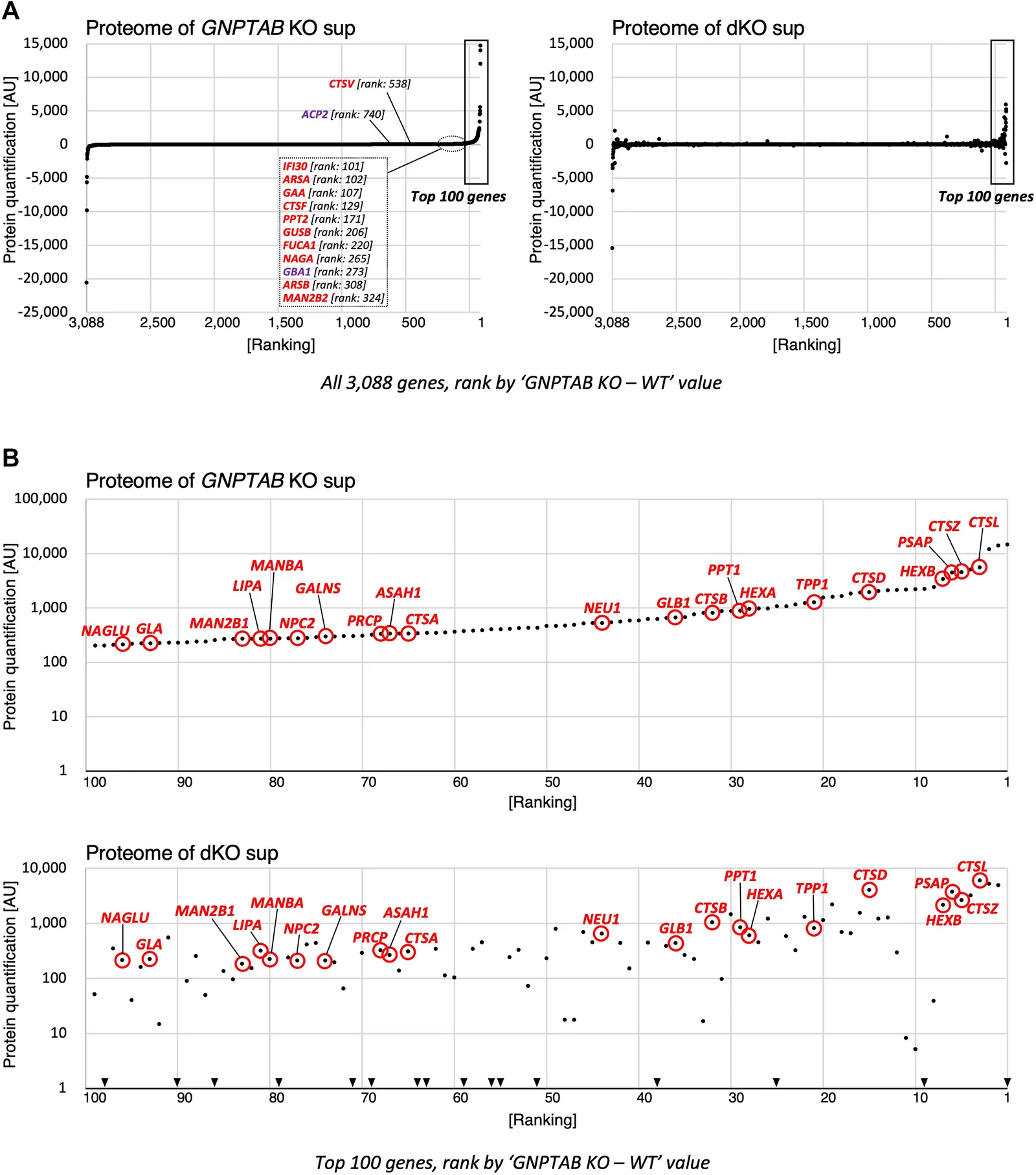
**Profile of secreted proteins in *GNPTAB* KO and dKO-culture supernatant.***A*, relative abundance of proteins detected in culture supernatants by proteomic analysis and shown as the difference obtained by subtracting the proteins present in the WT cell supernatant from those in the *GNPTAB* KO cell supernatant (*left panel*) and the dKO Sup supernatant (*right panel*), respectively. The proteins present in the supernatant of *GNPTAB* KO cells are listed in order of abundance (from *right* to *left*), along with their corresponding gene names. The proteins detected in the dKO supernatant are sorted in the same order. The top 100 ranked proteins are indicated by *rectangles*. 34 lysosomal proteins in total detected in the supernatants are shown. Lysosomal enzymes transported in an M6P-dependent manner are indicated in *red text*, while M6P-independent molecules are indicated in *blue text*. *B*, enlarged view of proteins ranked in the Top 100 in (*A*). Lysosomal enzymes are marked with a *red circle* and text. *Arrowheads* indicate the positions of proteins that yielded negative values in the difference calculation, where no lysosome-related molecules were included. M6P, mannose-6-phosphate.

### Supernatant of M6P receptor dKO cells (dKO sup) contains M6P-modified lysosomal enzymes capable of treating lysosomal diseases

The development of ERT for ML II and III patients has not been established. This is because, in these diseases, numerous lysosomal enzymes that rely on M6P for transport is simultaneously deficient, making it impractical to produce large quantities of lysosomal enzymes and replenish them all at once. Since M6P receptor KO is thought not to affect the M6P modification of lysosomal enzymes, the lysosomal enzymes secreted into the dKO sup are expected to be normally M6P-labeled. Since M6P-modified lysosomal enzymes could serve as a source for ERT for LSDs, including ML II, we next investigated the usability of the lysosomal enzymes in the dKO sup.

The dKO sup was collected after 2 days of incubation with regular fresh culture medium fetal bovine serum (FBS-containing DMEM) with these cells. Then, the *GNPTAB* KO cells were cultured with the dKO sup (hereafter, dKO sup treatment) for 24, 48, and 72 h. The increase in enzyme activity due to dKO sup treatment was observed in a time-dependent manner in *GNPTAB* KO cells, with some enzymes recovering to levels comparable to normal cells after 72 h of treatment (Fig. S2). According to this result, the dKO sup treatment was fixed at duration of 72 h (3 days) for further experiments. To examine the duration of the effects of enzyme supplementation, the culture medium was switched back from the dKO sup to regular culture medium after 3 days of dKO sup treatment, and the time course of withdrawal of lysosomal enzyme activities was observed. The increased enzyme activity by the dKO sup treatment gradually declined and returned to baseline levels for the *GNPTAB* KO cells within approximately 4 days (Fig. 3*A*). To confirm that the increase in lysosomal enzyme activity observed with dKO sup treatment was indeed mediated by M6P receptor-mediated enzyme uptake, we added various concentrations of M6P concurrently with dKO sup treatment and observed competitive inhibition effects. Compared to the untreated group (0 mM M6P), the addition of 5, 10, and 20 mM M6P inhibited lysosomal enzyme uptake into *GNPTAB* KO cells in a dose-dependent manner (Fig. 3*B*). These results indicate that dKO sup treatment resulted in the uptake of multiple M6P-modified lysosomal enzymes into *GNPTAB* KO cells *via* the M6P receptors.

**Figure 3:**
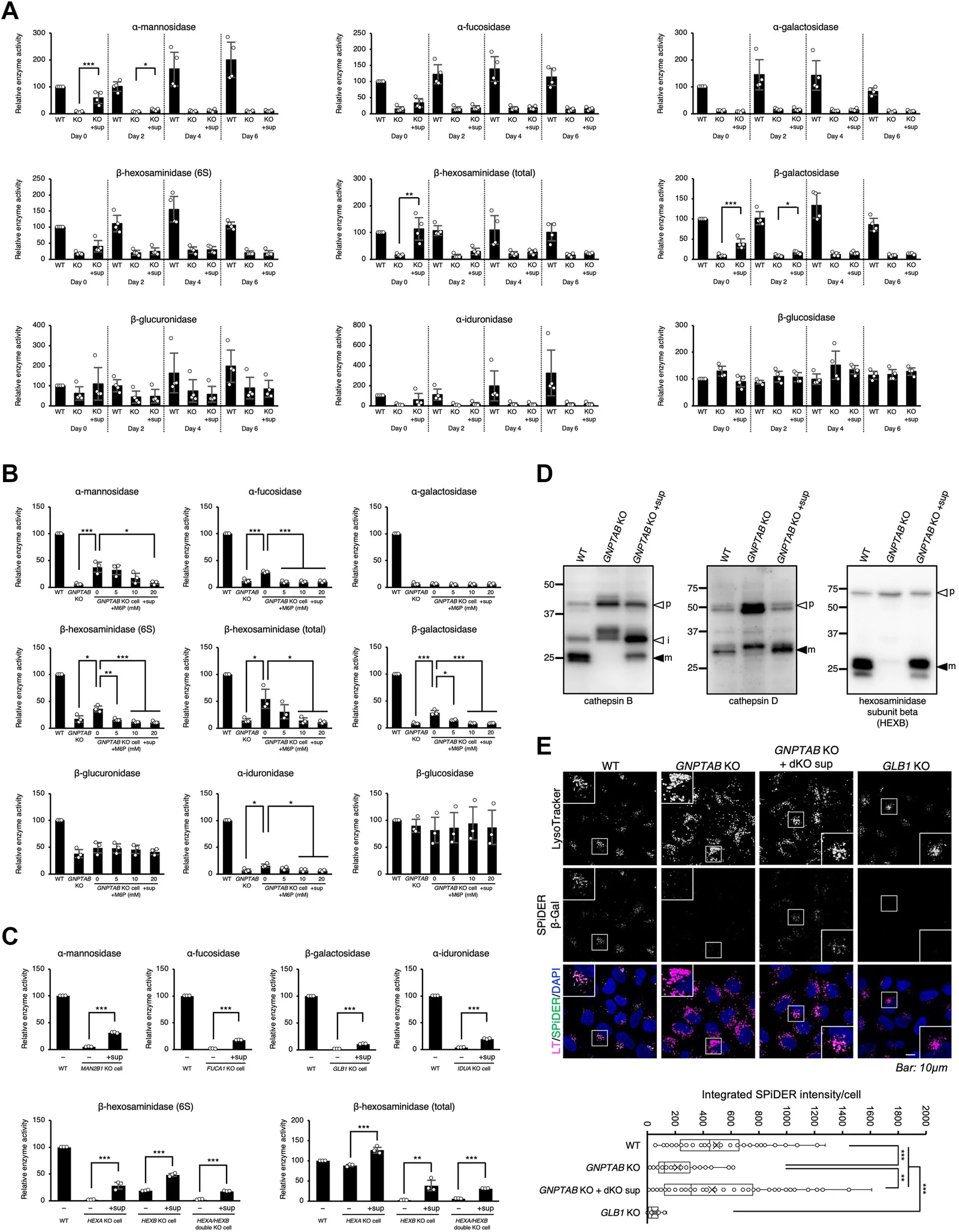
**Uptake of lysosomal enzymes by M6P receptors.***A*, uptake and withdrawal of lysosomal enzymes by *GNPTAB* KO cells treated with M6P receptors double KO supernatant. *GNPTAB* KO (indicated as KO) cell was cultured in a normal complete culture DMEM with/without M6P receptors double KO supernatant (1:1 mixture with DMEM, indicated as + sup) for 3 days (Day 0). After that culture medium was changed to a normal DMEM, and the indicated days were cultured (Days 2, 4, and 6). Relative values to WT lysosomal enzyme activities are shown (mean ± SD, n = 4). *B*, competitive inhibition of lysosomal enzyme uptake by M6P. *GNPTAB* KO cells were cultured with dKO sup for 3 days with 0, 5, 10, or 20 mM recombinant M6P. Cellular lysosomal enzymatic activities are shown (mean ± SD, n = 3). *C*, lysosomal enzyme uptake by LSD model cells. *MAN2B1*, *FUCA1*, *GLB1*, *IDUA*, *HEXA*, *HEXB* KO, and *HEXA* and *HEXB* double KO HeLa cells were treated with/without dKO sup for 3 days, and the lysosomal enzymes were measured. Relative activities to dKO sup nontreated WT cells are shown (mean ± SD, n = 3). The *white circles* represent data points from each independent experiment (*A* and *C*). *D*, western blotting analysis for the lysosomal enzyme processing. The cell lysates from WT, *GNPTAB* KO, and the dKO sup-treated *GNPTAB* KO cells were analyzed for cathepsin B (*left*), cathepsin D (*middle*), and the β-subunit of β-hexosaminidase (*right*). p: precursor, i: intermediate form, m: mature form. *E*, evaluation of *in situ* β-galactosidase activity with confocal imaging. The β-galactosidase activity in cells was visualized using the SPiDER probe. Enzyme activity was quantified as the integrated SPiDER signal overlapping with LysoTracker in each cell and plotted as a box-and-whisker plot. The box represents the upper quartile, median, and lower quartile; X marks indicate the mean values. Insets show magnified views of the boxed regions in each panel. The scale bar represents 10 μm. Statistical significance was assessed using *Student's t* test for (*A–C*) and the Kruskal-Wallis test for (*E*) (∗*p* < 0.05, ∗∗*p* < 0.01, ∗∗∗*p* < 0.005). dKO, double KO; M6P, mannose-6-phosphate.

Furthermore, we confirmed that dKO sup treatment has the potential to restore enzyme activities in other lysosomal enzyme-deficient cells besides *GNPTAB* KO, *MAN2B1* KO cells (a model for α-mannosidosis), *FUCA1* KO cells (fucosidosis), *GLB1* KO cells (GM1 gangliosidosis), *IDUA* KO cells (mucopolysaccharidosis type I), *HEXA* KO cells (Tay-Sachs disease), and *HEXB* KO cells (Sandhoff disease) (Fig. 3*C*) that were established by genome editing using the CRISPR/Cas9 method (Table S1). These results suggest that the dKO sup can broadly treat pathological conditions caused by the dysfunction of the individual enzymes that are transported in an M6P-dependent manner.

Since the activity of lysosomal enzymes is measured using cell lysates, it is not possible to verify whether the enzymes taken up into the cells from the dKO sup are properly transported to the lysosomes, processed, and exhibit enzymatic activity. Therefore, we examined the processing level of cathepsin B, cathepsin D, and the hexosaminidase β subunit by Western blotting using cell lysates from WT, *GNPTAB* KO, and *GNPTAB* KO cells treated with dKO sup. The results showed that in *GNPTAB* KO cells, many samples exhibited bands corresponding to precursor (p) or intermediate (i) forms, or irregular bands thought to result from abnormal glycosylation. In contrast, in *GNPTAB* KO cells treated with dKO sup, bands corresponding to the mature (m) forms of all three enzymes were observed, suggesting that enzymes taken up from the extracellular space were properly processed in the acidic environment of the endosomes/lysosomes (Fig. 3*D*). Furthermore, we examined intracellular β-galactosidase activity using the SPiDER probe, which can detect lysosomal enzyme activity *in situ*. The signal from the SPiDER-β-Gal probe colocalized with LysoTracker, and the SPiDER signal in *GNPTAB* KO cells was significantly weaker than that in WT cells. When *GNPTAB* KO cells were treated with the dKO sup, a significant recovery of the SPiDER signal was observed, and these signals colocalized with LysoTracker. These results support the notion that β-galactosidase in dKO sup is taken up by *GNPTAB* KO cells, properly transported to the lysosomes, and exerts enzymatic activity (Fig. 3*E*).

### dKO sup treatment restores cellular function in GNPTAB-deficient cells

Autophagy is an essential process for maintaining cellular homeostasis by degrading obsolete proteins and organelles that have lost their proper functions within the cell, and it is dependent on the lysosomal degradative functions (25, 26, 27). In the ML II patient cells, autophagic function is significantly impaired due to reduced lysosomal function (22, 28). Similarly, in *GNPTAB* KO cells, the compromised lysosomal functions lead to a significant decrease in autophagic activity compared with WT, gene non-edited HeLa cells. When *GNPTAB* KO cells were treated with the dKO sup for three days, autophagic activity was significantly restored in both nutrient-rich and starved conditions (Fig. 4*A*, upper). After removal of the dKO sup, the improved autophagic activity returned to the original level of *GNPTAB* KO cells by the fourth day, in accord with the reduction of the lysosomal enzyme activity (Fig. 4*A*, lower).

**Figure 4:**
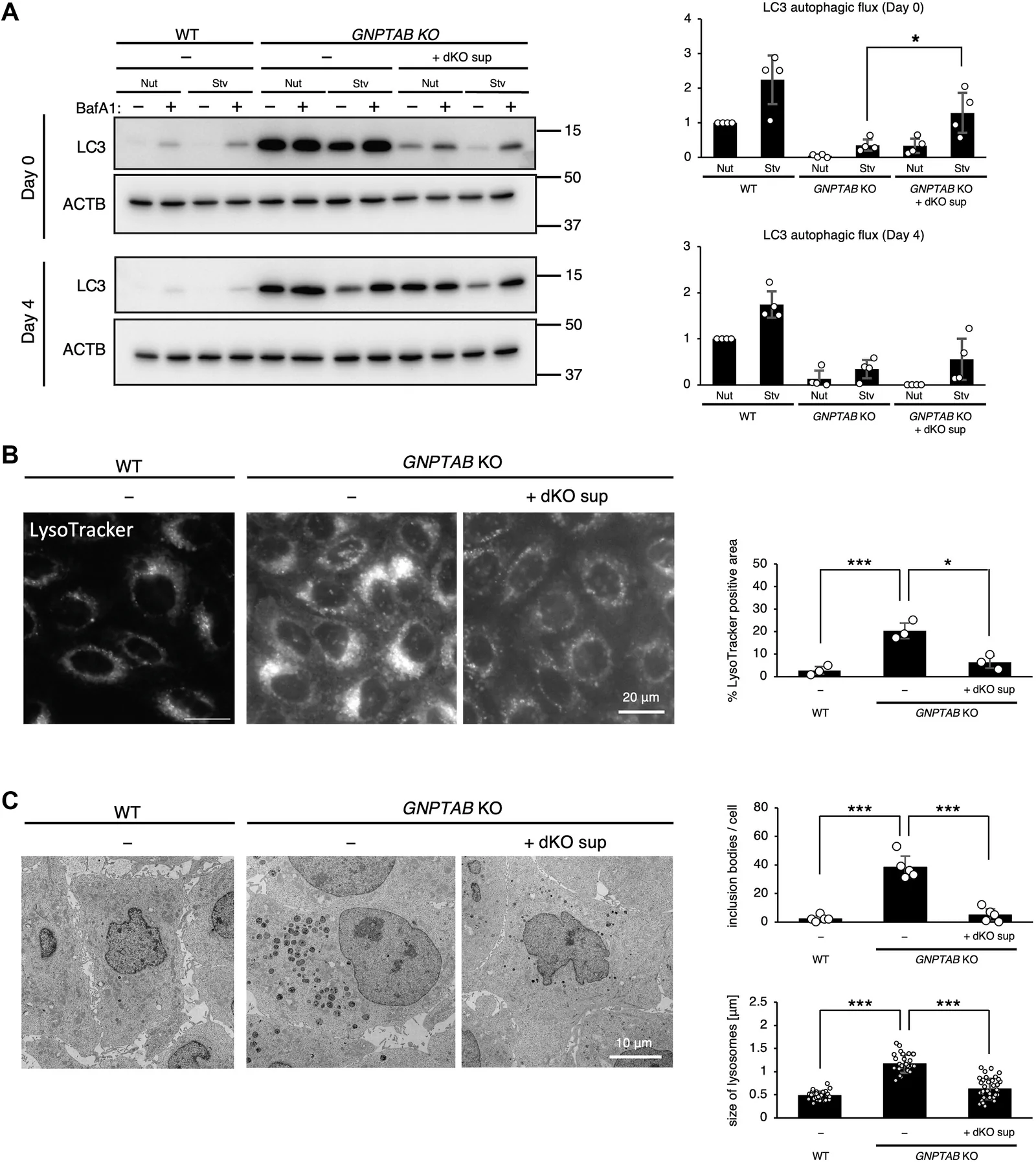
**Restoration of cellular function by dKO sup treatment in *GNPTAB* KO cells.***A*, restoration of autophagic function, (*B*) lysosomal function, and (*C*) cellular morphology. *GNPTAB* KO cells were treated with dKO sup for 3 days and subjected to the LC3 autophagic flux assay, LysoTracker staining, and electron microscopy investigation. For the autophagic flux assay, cells were incubated for 2 h under nutrient-rich (complete DMEM) or starvation (EBSS) conditions, in the presence or absence of a lysosomal inhibitor, bafilomycin A1 (BafA1). LC3 flux was calculated based on the difference between conditions with and without BafA1 and then normalized to the LC3-II levels under the condition without BafA1. LysoTracker staining was performed with 100 nM for 30 min. LysoTracker-positive regions were quantified and normalized to the total cell area (n = 4). Using an electron micrograph, the number of intracellular inclusion bodies was counted (n = 5). *Error bars* indicate ± SD. The *white circles* represent data points from each independent experiment (*A* and *B*), from the number of inclusion bodies in each cell (*C*, *top*), or from the size of each lysosome (*C*, *bottom*). Significance was calculated with Student's *t* test (∗*p* < 0.05, ∗∗∗*p* < 0.005). dKO, double KO.

LysoTracker staining represents stronger signals in the *GNPTAB* KO cells compared to the WT cells, reflecting abnormal lysosome accumulation containing undigested materials (21, 22, 28, 29). The enhanced LysoTracker signal was reversed to almost normal levels upon the dKO sup treatment (Fig. 4*B*). Similarly, an electron microscopic analysis revealed the accumulation of undegraded substances (*i.e.*, "inclusion bodies") and a 2–3-fold increase in lysosomal diameter in the *GNPTAB* KO cells, which can be attributed to impaired lysosomal degradation. Notably, these accumulated materials were largely eliminated following the dKO sup treatment (Fig. 4*C*). Altogether, these findings suggest that the dKO sup can effectively restore intracellular abnormalities associated with lysosomal dysfunction in the ML II.

### The dKO sup treatment restores cellular function in skin fibroblasts derived from ML II patients and improves their viability rate after cryopreservation

ML II patient fibroblasts exhibit a swollen morphology and are characterized by numerous inclusion bodies in the cytoplasm under phase-contrast microscopy observation. These phase-dense bodies became the origin of the disease name inclusion cell disease (I-cell disease) (30, 31, 32). When ML II skin fibroblasts (SF) were subjected to dKO sup treatment for three days, a significant recovery in the activity of lysosomal enzymes was observed, as shown in the HeLa model cells (Fig. 5*A*). Furthermore, the abnormal accumulation of cholesterol, which is markedly evident in the ML II SF, was resolved by the dKO sup treatment (Fig. 5*B*). Along with this restoration of cellular function, the morphology of the ML II SF was much improved, adopting the typical elongated shape of normal skin fibroblasts without inclusion bodies (Fig. 5*C*).

**Figure 5:**
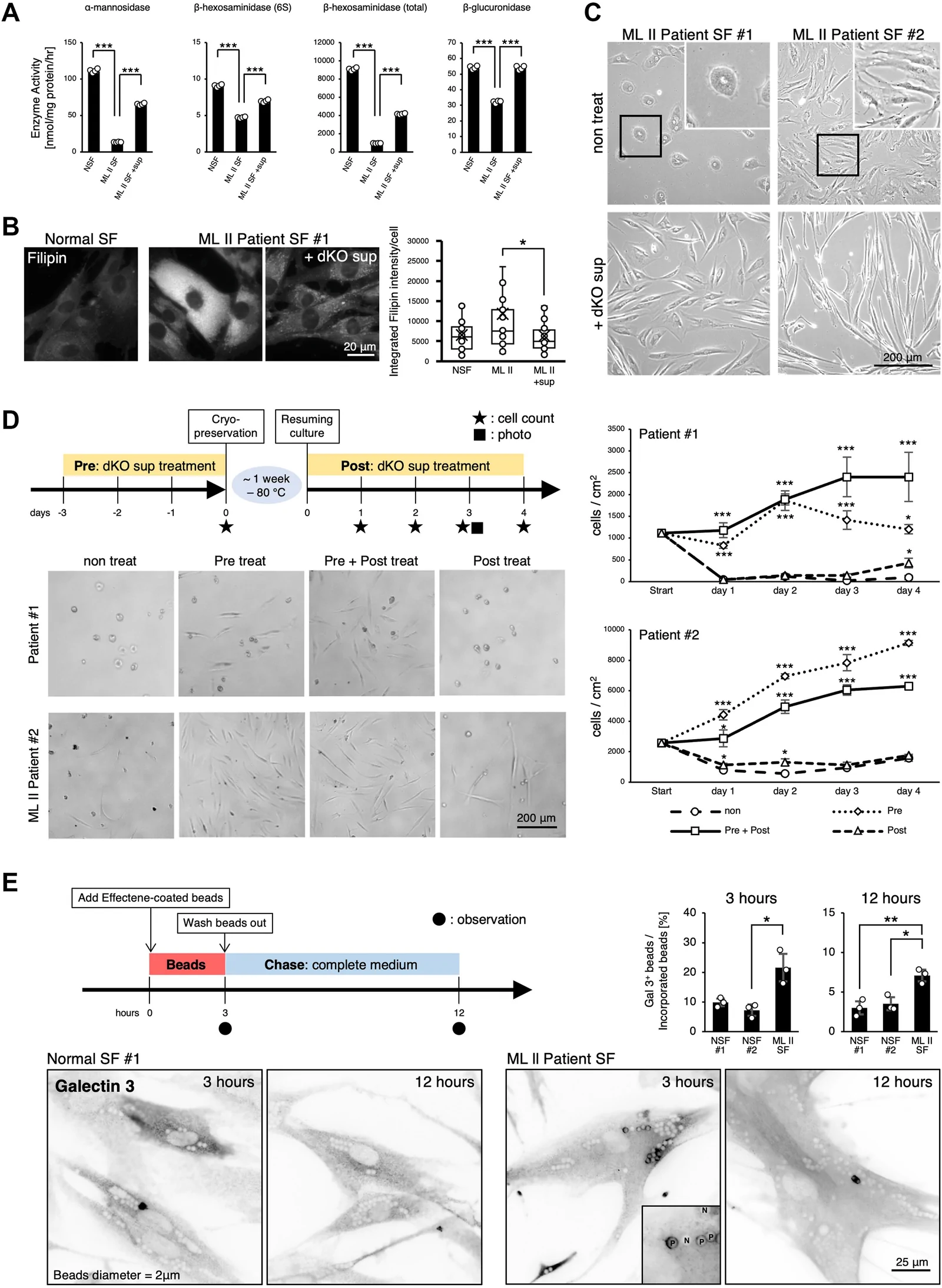
**Characteristic change of ML II patient skin fibroblasts by dKO sup treatment.***A*, measurement of lysosomal enzyme activity in patient-derived skin fibroblasts treated with dKO sup. The enzymes measured are indicated at the *top* of *each panel*. Normal Skin Fibroblasts. ML II SF: Skin Fibroblasts from an ML II patient #1. *B*, imaging analysis of cholesterol accumulation in fibroblasts from an ML II patient. Intracellular free cholesterol was visualized using Filipin III, and the integrated intensity per cell was quantified. *C*, morphologic change. ML II patient skin fibroblasts were cultured with/without dKO sup for 3 days, and cell morphology was observed. *D*, improvement of cryo-viability. ML II patient skin fibroblasts were treated with/without dKO sup for 3 days before cryopreservation. One week later, cells were recultured with/without dKO sup, and the number of surviving cells was counted up to 4 days after resuming culture. Pre: culture with dKO sup for 3 days before cryopreservation. Post: culture with dKO sup after resuming culture. Non: without dKO sup treatment. The bar graph indicates mean ± SD (n = 3). *E*, vulnerability of ML II lysosomal membrane. Two Normal SF and 4 ML II SF lines were tested independently. Effectene-coated beads were internalized for 3 h, and lysosomal membrane rupture was tracked at 0 and 9 h. Percentages of lysosomal rupture, [Gal3-positive beads]/[Total incorporated beads], were calculated from 10 images across different fields of view. 309 to 1395 beads were analyzed in each group. N and P in the inset indicate Galectin 3-negative and -positive beads, respectively. The *white circles* represent data points from each independent experiment (*A*, *D* and *E*), or Filipin signal intensity of each cell (*B*). The statistical significance was calculated with One-way ANOVA followed by Tukey’s *post hoc* test for (*A*), with the Kruskal-Wallis test followed by the Mann-Whitney U test with Holm’s correction for (*B*), with Student's *t* test for (*D*), and Welch’s *t* test for (*E*) (∗*p* < 0.05, ∗∗∗*p* < 0.005). dKO, double KO.

A characteristic feature of patient-derived ML II cells is their extremely low viability after cryopreservation, which often poses an obstacle in ML II research (33, 34). The viability of the untreated ML II SF after a freeze-thaw cycle was less than 5% of the input before cryopreservation. In contrast, ML II SF treated with the dKO sup before freezing showed a significant improvement in cell viability after thawing (Fig. 5*D*). We further tested the effects of dKO sup treatment on the viability by adding it immediately after thawing, but the cell viability did not show any significant changes from that of untreated specimens. We also tested both pre-freeze and post-thaw dKO sup treatments, and the results were similar to cells treated only with the pre-freeze treatment (Fig. 5*D*). Taking these results together, it is important to improve the condition of cells by treating them with dKO sup before cryopreservation, which may confer resistance against the physical stress of freezing, and no additional effect is expected from post-thaw treatment.

We additionally confirmed whether ML II lysosomes are fragile. We coated beads with a non-liposome-based lipid transfection reagent used for DNA transfection and allowed them to be internalized *via* endocytosis. Beads internalized into the endo-lysosome system permeabilize the vesicle membrane. When the lysosomal lumen, containing sugar-rich glycoproteins like Lamp1/2, communicates with the cytoplasm, the cytoplasmic lectin galectin-3 (Gal3) accumulates on the sugar chains within the lysosome. Gal3-positive vesicles can be identified as vesicles with ruptured membranes (35, 36, 37). In ML II patient cells, a higher proportion of lysosomal membranes were observed to be ruptured compared to WT (Fig. 5*E*). This leads to the conclusion that lysosomes in ML II are fragile. This finding may provide one explanation for the low cryo-viability observed in ML II.

These results indicate that mucolipidosis cells exhibit abnormalities in the composition of plasma membranes, and that treatment of Mucolipidosis cells with the dKO sup restores normal plasma membrane dynamics through improving lysosomal functions.

### The dKO sup treatment restores cellular function in *NPC2*-deficient cells

Abnormal metabolism of cholesterol is known in LSDs such as ML II, LAL deficiency, and Niemann-Pick disease. Next, to more broadly validate the efficacy of the dKO sup treatment, we investigated disease model cells with the KO of cholesterol metabolism-related genes.

As reported in the patient-derived SF (28, 38), intracellular cholesterol levels were higher in *GNPTAB* KO cells compared to WT cells. The treatment of *GNPTAB* KO cells with the dKO sup significantly reduced cholesterol accumulation (Fig. 6*A*). Consistent with this, fluorescent microscopic analysis also revealed that the intracellular free cholesterol visualized by Filipin III (39) was also reduced after the treatment with the dKO sup (Fig. 6*B*).

**Figure 6:**
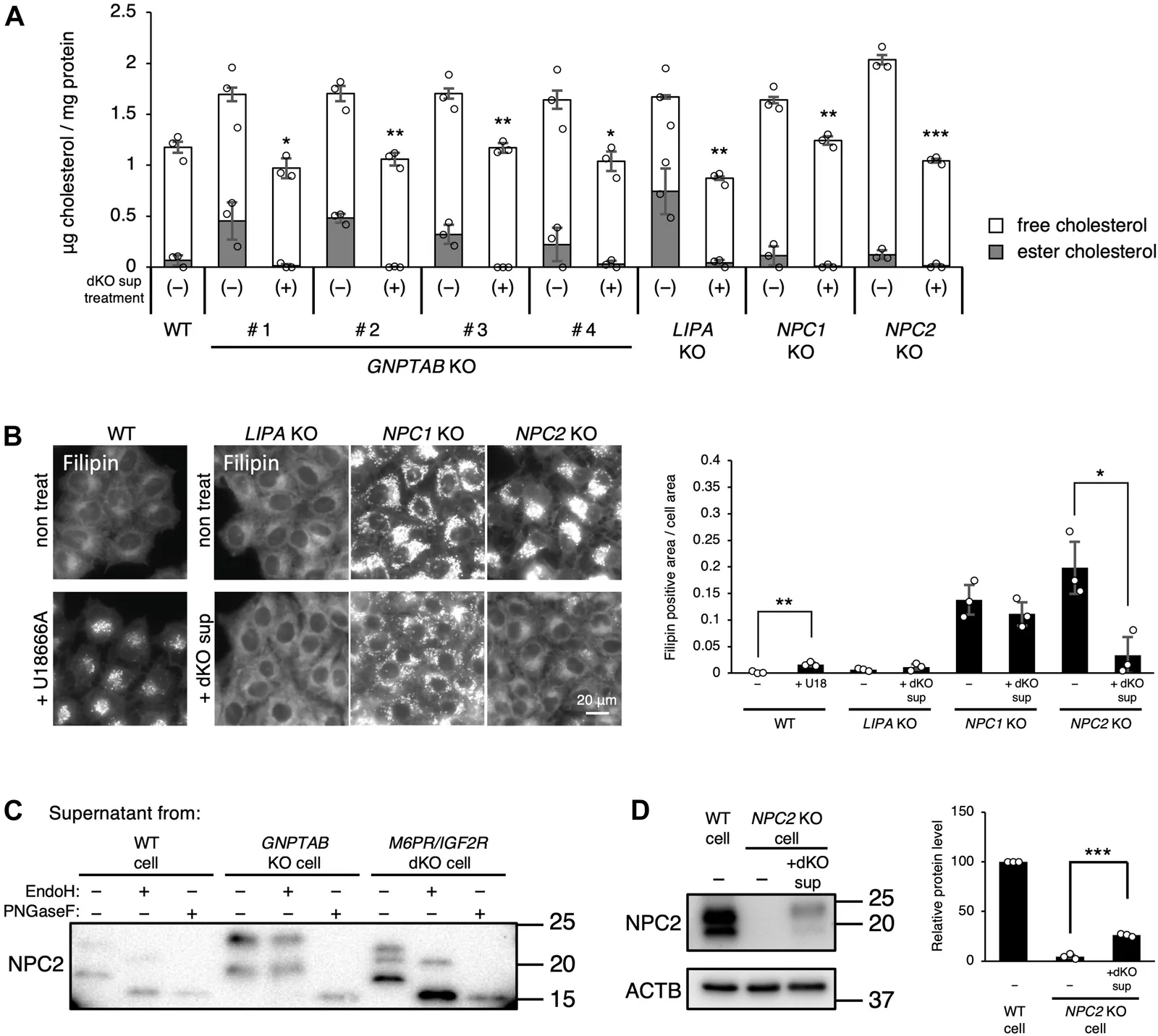
**Restoration of cellular function by dKO sup treatment in cholesterol *NPC2* KO cells.***A*, improvement in cholesterol accumulation. *GNPTAB*, *LIPA*, *NPC1*, and *NPC2* KO cells, as well as WT HeLa cells, were cultured in conventional complete DMEM or dKO sup medium for 3 days, and intracellular cholesterol levels were measured. Both free and esterified cholesterol were presented in a stacked column chart as the sum of free (*top*) and esterified (*bottom*) cholesterol (mean ± SD, n = 3). *B*, unesterified cholesterol (Filipin) staining. WT cells and *LIPA*, *NPC1*, and *NPC2* KO cells were treated with 2 μg/ml U18666A for 24 h or dKO sup for 3 days. Then the cells were stained with Filipin III. Filipin-positive area was quantified and expressed as a percentage of the total cellular area (mean ± SD, n = 3). *C*, NPC2 protein secreted in supernatants. The culture supernatants from the indicated cells were collected, treated with/without glycosidases, and then the NPC2 protein was detected by immunoblotting. *D*, supplementation of NPC2 protein in *NPC2* KO cells by dKO sup. Bar graph indicates mean ± SD (n = 3). The white circles represent data points from each independent experiment (*A*, *B* and *D*). The significance between the dKO sup untreated and treated samples was calculated using Student's *t* test (∗*p* < 0.05, ∗∗*p* < 0.01, ∗∗∗*p* < 0.005). dKO, double KO; NPC2, Niemann-Pick disease type C2.

In addition to *GNPTAB* KO cells, *LIPA*, *NPC1*, and *NPC2* KO cells were established (Table S1). The *LIPA* gene encodes LAL, a primary enzyme responsible for hydrolyzing cholesteryl esters and triglycerides within lysosomes (40), which is also one of the representative hydrolases that is transported to lysosomes *via* the M6P-dependent pathway (41). The accumulation of esterified cholesterol observed in *GNPTAB* KO and *LIPA* KO cells can be attributed to the failure of proper transport of LAL to the lysosomes, resulting in impaired hydrolysis of esterified cholesterol. dKO sup treatment reduced the accumulation of esterified cholesterol in these cells (Fig. 6*A*), suggesting that M6P-modified LAL was included in the dKO sup and supplied to the lysosomes of the recipient KO cells.

NPC, caused by mutations in the *NPC1* or *NPC2* genes, is characterized by the accumulation of free cholesterol and other lipids in the endo/lysosomal compartments (42). NPC2 binds and transfers cholesterol to the luminal side of the lysosomal transmembrane protein NPC1, and NPC1 facilitates the transport of cholesterol across the lysosomal membrane to other cellular compartments. This cooperative process is essential for maintaining cellular lipid balance (43). Filipin III staining confirmed the free cholesterol accumulation in both *NPC1* and *NPC2* KO cells (Fig. 6*B*). NPC is not a disease caused by enzyme abnormalities but rather by defects in lipid transport proteins. The dKO sup treatment successfully reduced the accumulated cholesterol in *NPC2* KO cells. In contrast, no significant intracellular cholesterol clearance was observed in *NPC1* KO cells (Fig. 6*B*). It has already been reported that adequate M6P modification is necessary for the lysosomal targeting of NPC2 (44). M6P-modified NPC2 protein is also secreted extracellularly from the M6P receptor dKO cells (Fig. 2), and it suggests that the addition of the dKO sup treatment could replenish functional NPC2 in the *NPC2* KO cells. To test this possibility, NPC2 protein levels in the culture supernatants of WT, *GNPTAB* KO, and *M6PR*/*IGF2R* dKO cells were examined by western blotting. The results showed that highly shifted bands of NPC2 at molecular size, compared with WT cells, which were EndoH resistant and PNGaseF sensitive, were detected in *GNPTAB* KO cells (Fig. 6*C*), indicating that NPC2 protein modified with complex-type sugar is being secreted extracellularly due to GNPT deficiency. Conversely, in M6P receptor dKO cells, larger amounts of NPC2 of the same size as WT cells were secreted. Furthermore, *NPC2* KO cells treated with dKO sup showed restored intracellular NPC2 levels (Fig. 6*D*). These results support the validity of our hypothesis for NPC2 delivery.

## Discussions

Our current study revealed that depletion of M6P on the ligand side (lysosomal enzymes) and depletion of the M6P receptor on the receptor side produce similar enzyme activity profiles in their culture supernatants and endo-lysosomal structures. While the phenotypes in lysosomal enzymatic activities were clear when both M6PR and IGF2R were depleted, the effect of *M6PR* KO was generally milder than that of *IGF2R* KO. Furthermore, for some enzymes like α-fucosidase, dependence on IGF2R was relatively high; depletion of IGF2R reduced activity in cells to only about one-quarter of that in normal cells. These observations are consistent with the previous report (45) and support their conclusion that M6PR and IGF2R do not exhibit complete functional redundancy in lysosomal enzyme transport. Differences in enzyme activities of the supernatant and inside the cell between the *M6PR* and *IGF2R* single KO may reflect their distinct affinities for individual lysosomal enzymes. The precise differences in affinity for M6P receptors between individual lysosomal enzymes represent a crucial issue not only for understanding disease pathogenesis but also for the development of enzyme replacement therapy. Further studies are needed.

In this report, we established the *M6PR*/*IGF2R* dKO cell supernatant (the dKO sup) as a source of M6P-modified lysosomal enzymes, which can be used to restore lysosomal function in various types of LSDs. Although the degree of recovery of activities varies by enzyme, ranging from about 10% to complete recovery of WT levels, previous reports have shown that approximately the remaining 10% enzymatic activity was sufficient to prevent disease onset for some lysosomal disorders (46, 47), suggesting the therapeutic potential of the dKO sup would be sufficient for ERT. The approach of the single or double-depletion of the M6P receptors is not only applicable for developing efficient enzyme production methods in ERT, but also for *ex vivo* genetically modifying donor cells to efficiently release enzymes extracellularly during hematopoietic stem cell transplantation.

The potential for ERT in ML II and III has already been demonstrated (28). In that report, a lysosomal enzyme mixture was collected from the culture supernatant of normal cells treated with ammonium chloride (NH_4_Cl). NH_4_Cl disrupts the intracellular membrane transport environment, facilitating the leakage of M6P-modified lysosomal enzymes into the culture supernatant. While this method requires removal of NH_4_Cl from the culture medium, the M6PR dKO strategy presented in the present study eliminates the need for NH_4_Cl removal steps *via* dialysis or ultrafiltration, making it highly convenient and enabling continuous, higher-efficiency enzyme recovery from the production cells. In ML II or III, which is caused by GNPT deficiency, multiple lysosomal enzymes are deficient within cells, and the single-enzyme ERT strategy currently attempted for LSDs such as Fabry disease, Gaucher disease, and mucopolysaccharidoses cannot be applied to ML II or III. As GNPT is a hexameric protein complex encoded by the two genes and is composed of 2 sets of three subunits after intracellular processing and assembly (14, 20), this makes it difficult to supply active GNPT enzyme to the proper subcellular location where this enzyme functions from outside the cell. Therefore, the approach of the dual depletion of M6P receptors demonstrated here could become a powerful tool for the treatment of ML II and III.

Although the precise reason why ML II cells fail to survive cryopreservation remains unclear, lysosomal dysfunction could alter lipid or protein composition within plasma and organelle membranes, which can compromise their integrity through several mechanisms. An impaired intracellular membrane trafficking reported in ML II cells (28) may be responsible for the reduced plasma membrane fluidity and self-repair capacity (48). Lysosomal enlargement and membrane destabilization can trigger lysosomal membrane permeabilization, leading to the release of lysosomal enzymes such as cathepsins into the cytosol. lysosomal membrane permeabilization -induced cathepsin release has also been implicated in the initiation of both apoptosis and necrosis (49, 50). Collectively, these combined defects may render cells more vulnerable to external stresses, including freeze-thaw cycles. The dKO sup treatment is not effective unless it is performed before freezing ML II cells. To enhance viability after cryopreservation, it is necessary to improve the cellular pathology, possibly by eliminating accumulated substances and normalizing lipid composition before freezing. This enables the preservation and utilization of valuable research resources like ML II patient-derived skin fibroblasts. In addition, the results in Figure 3 suggest that the enzyme activity levels in the cells return to those of the untreated diseased cells more than 4 days after thawing and resumption of culture. These cells can be used for pathological analysis of *GNPTAB* KO or ML II after a certain period of subculture. This method for achieving reversible, temporary recovery of lysosomal functions has a significant practical advantage in research in this field.

Although treatments such as cyclodextrin, as therapeutics aimed to reduce accumulated cholesterol in NPC, are currently in trials (51, 52, 53, 54, 55, 56), only symptomatic treatments are available for this disease. Previous reports have shown that the NPC2 protein reduces cholesterol accumulation in fibroblasts from NPC2 patients (57, 58, 59) and ameliorates the pathological condition in NPC2 KO mice (60). Our study demonstrates that the NPC2 protein from human resource is properly modified and actually taken up by cells, and then reduces cholesterol accumulation, which clearly establishes the therapeutic concept of protein replacement therapy. These findings open the door to an entirely new potential curative strategy for NPC2. However, it is important to note that our research findings are merely a proof of concept and cannot be directly linked to clinical applications. In particular, as with conventional ERT, delivering treatment to the central nervous system and bones will likely remain challenging. Furthermore, to maintain long-term intracellular enzyme activity, it will be necessary to explore optimization strategies, such as concentrating and purifying the M6P-modified protein or increasing the dosage. Moving forward, it is necessary to investigate in greater detail the effects of the dKO sup on cellular metabolic systems and to explore its potential for clinical application through *in vivo* therapeutic experiments using animal models of ML II, LAL deficiency, and NPC disease.

## Experimental procedures

### Cell lines and cell culture

HeLa-Kyoto cells (referred simply to as HeLa cells in this manuscript), established as a sub-line of the original HeLa cells at Kyoto University, are now globally distributed without any limitation, and we obtained them through EMBL. Normal human skin fibroblasts were obtained from Gibco (Thermo Fisher Scientific), Lonza, and Kurabo. The skin fibroblast lines derived from mucolipidosis patients were established from the remaining specimens for diagnostic or other research purposes for which written informed consent was obtained. In Figure 5, *A*–*D*, Pathogenic variants in the *GNPTAB* gene in ML II patients #1 and #2 were c.310C4T (p.Q104X)/c.2544delA (p.K848fs) and c1120T>C (p.F374L)/c.3565C>T (p.R1189X), respectively. As for the patient-derived skin fibroblasts used in Figure 5*E*, they were clinically diagnosed, but their genotypes were not determined. The use of the patients’ specimens was approved by the institutional ethics review boards (Osaka University: 703 and Kawasaki Medical School: 5889). The human subject research reported in this manuscript complies with the principles of the Declaration of Helsinki. HeLa cells and the skin fibroblasts were maintained at 37 °C in a humidified 5% CO_2_ incubator in high-glucose DMEM (D5796; Merck) supplemented with 10% FBS (173012; Merck) and penicillin/streptomycin (100 units/ml and 100 μg/ml, respectively; 09367-34; Nacalai Tesque, Japan) for normal growth conditions (nutrient-rich). For nutrient starvation, cells were cultured in EBSS (E2888; Merck). All cell lines used this study were confirmed as *mycoplasma* free.

### Antibodies

Primary antibodies used were: β-actin (1:5000, M177-3; MBL), LC3 (1:1500, PM036; MBL), NPC2 (1:1000, 19888-1-AP; Proteintech, IL), and Gal3 (1:1000, sc-23938), cathepsin B (1:1000, AF953, R&D, MN), cathepsin D (1:1000, AF1014, R&D), hexosaminidase β subunit (1:1000, sc-376781). horseradish peroxidase -conjugated secondary antibodies used for immunoblotting were: Goat anti-rabbit IgG (31460, Thermo Fisher Scientific) and Goat anti-mouse IgG (31430, Thermo Fisher Scientific).

### Reagents

Commercially available compounds were obtained as follows and used in this study: Bafilomycin A1 (BafA1; 11038, Cayman Chemicals), Mannose-6-phosphate (M6P; M6876, Merck), U18666A (218-01441, Fujifilm-Wako). 4MU-conjugated lysosomal enzyme substrates for β-glucosidase (M3633, Merk), β-hexosaminidase (M2133, Merk), α-mannosidase (M3657, Merk), α-fucosidase (M8527, Merk), α-galactosidase (M7633, Merk), β-galactosidase (M1633, Merk), and β-glucuronidase (M9130, Merk), α-hexosaminidase (44009, Glycosynth), α-iduronidase (44076, Glycosynth).

### Generation of KO cells

pSpCas9 (BB)-2A-GFP (px458) was a gift from Feng Zhang (Addgene plasmid #48138). Guide RNAs were designed using the Benchling CRISPR Guide RNA Design Tool (https://www.benchling.com/crispr/) and cloned into the px458 vector. The guide sequences for establishing each KO cell are as follows; *GNPTAB*: 5′ ACACG TAGAG CCCAT ACCTG 3′, *M6PR*: 5′ GAAGC TGGCA ACCAC ACTTC 3′, *IGF2R*: 5′ GACAC GCACT TATCA TTCAG 3′, *MAN2B1*: 5′ ACGTA AATGA AGCGA CGGGT 3′, *FUCA1*: 5′ GAACT TGGCT TCGTC GAACC 3′, *GLB1*: 5′ CCCGTG TGCCC CGCTT CTAC 3′, *HEXA*: 5′ GTTGT CTCTG TAGTC ACACC 3′, *HEXB*: 5′ GAGGG GCCCG CCGTG GAATT 3′, *IDUA*: 5′ GCTCC TCCAG AAGCG CCGCA 3′, *LIPA*: 5′ TCCCA TGAGG AATTC GGTTA 3′, *NPC1*: 5′ GCGCT GGACA CAGTA GCAGC 3′, *NPC2*: 5′ GTTAT CCGCG ATGCG TTTCC 3′, *GNPTG*: 5′ CACGG GTGAA GGATC CCTCT 3′, *NAGPA*: 5′ GAGAC GACGC GCCAC CGTGG 3′. Cells were transfected with the targeting px458 vector using Effectene (301425; Qiagen) or PEI Max (24765; PolySciences, IL). After 48 h of transfection, GFP-positive cells were single-cell sorted using BD FACS Aria III Cell Sorter (BD Biosciences) into 96-well plates and cultured for 2 weeks. Genomic DNA was extracted using the QuickExtract DNA extraction solution (QE0905T; Lucigen), and the target loci were analyzed by Sanger sequencing after PCR amplification of the 300 to 400 bp region surrounding the targeted site. PCR amplification was performed using the forward (Fw) and reverse (Rv) primers listed below. Cycle Sequencing was then conducted using the same Fw primers. *GNPTAB*: 5′ CGCCG GAGCT GCAAT GAGCG 3′ (Fw) and 5′ ATCGC CTGAG CTCAG GAGTT CG 3′ (Rv), *M6PR*: 5′ ATCGA GCATG TTGTT TGTTT 3′ (Fw) and 5′ ATTAG GCATC CTGTG GTATA TTA 3′ (Rv), *IGF2R*: 5′ AAGCA AATGT GGCTT GAATA 3′ (Fw) and 5′ CTAGA CAGAA CCAAC CTAGT 3′ (Rv), *MAN2B1*: 5′ ATGGT CCCTG TAATC CCTCT 3′ (Fw) and 5′ GGAGC GACAC GCATG TTATA 3′ (Rv), *FUCA1*: 5′ CGGGC CAATC GTTAG TCAGA 3′ (Fw) and 5′ GAAGA AGCGC GCAGT GAACT 3′ (Rv), *GLB1*: 5′ GCATT AAGTC CATCA GCACA 3′ (Fw) and 5′ CAGAA CATCA CACTG GACCA 3′ (Rv), *HEXA*: 5′ CACAA CGAGA GTGAG ACAGC 3′ (Fw) and 5′ TTAGC ACTGG TTAAG TTTCT GC 3′ (Rv), *HEXB*: 5′ TGCTG ACTCA GGTGG CGCTG 3′ (Fw) and 5′ TCCCT CTTCC ACTTA CTATC CGGC 3′ (Rv), *IDUA*: 5′ ACTCC GACCC GGAGG CGGAA 3′ (Fw) and 5′ GGAGA CTCCT GGTTT ACCTC 3′ (Rv), *LIPA*: 5′ GGGAG GATTT ACTCT TGTGA TG 3′ (Fw) and 5′ CTTTG CCTTG CAAAC CTCAA 3′ (Rv), *NPC1*: 5′ AACAC GGGGA CCTTG AAGCG 3′ (Fw) and 5′ CATCC TACAC AGAGA AATTA GGTGA 3′ (Rv), *NPC2*: 5′ GCCGG AGAGG TGCTC TAAAG 3′ (Fw) and 5′ AGAAA CAAAG TTGTG CGCGG 3′ (Rv), *GNPTG*: 5′ GCTCA CGGTC TCGCT CCCC 3′ (Fw) and 5′ TTGCA TAGAC ACGCA CGTAA AAG 3′ (Rv), and *NAGPA*: 5′ GAGCA CGAGA GTTGG CCTCC 3′ (Fw) and 5′ CAAAT CTCAG GCCCT CTGCC C 3′ (Rv).

### Measurement of lysosomal enzyme activity

The 4-MU enzyme assay was performed as described previously (61). Cell lysates (“cell”) prepared at a protein concentration of 2 mg/ml, or 10 μl of culture supernatant (“sup”), were used for the reactions. For measurement of total β-hexosaminidase activity, cell lysates were diluted to 20 μg/ml. Samples were incubated with artificial substrates in acidic phosphate or citrate buffer at 37 °C for 1 h, and fluorescence was measured at Ex/Em 365/450 nm using a microplate reader (Infinite 200 PRO; TECAN). Enzyme activities are described as nmol/mg protein/h based on 4-MU standards.

### Proteome analysis of cell culture supernatants

Serum-free culture supernatants from WT, *GNPTAB* KO, *M6PR* KO, *IGF2R* KO, and *M6PR*/*IGF2R* dKO cells were obtained by culturing the cells in OPTI-MEM (31985062, Thermo Fisher Scientific) for 24 h. Proteomic analysis of the collected supernatants and control medium was performed by Creative Proteomics. Protein samples were subjected to 4D-direct DIA proteomic analysis. Proteins were extracted in 8 M urea containing protease inhibitors and quantified using a bicinchoninic acid assay. The samples were digested with trypsin using an iST Sample Preparation Kit (PreOmics), followed by peptide cleanup according to the manufacturer’s instructions. The resulting peptides (200 ng) were analyzed on a Vanquish Neo UHPLC system coupled to a timsTOF HT mass spectrometer (Bruker Daltonics). The Peptides were separated on an IonOpticks Aurora C18 column (75 μm × 8 cm, 1.7 μm particles) using a 30-min gradient at a flow rate of 300 nl/min. DIA data were acquired in diaPASEF mode. Raw data were processed using Spectronaut version 20 (Biognosys), and spectra were searched against the UniProt human database (https://www.uniprot.org/). Carbamidomethylation of cysteine was set as a fixed modification, whereas oxidation of methionine and protein N-terminal acetylation were set as variable modifications. Trypsin was specified as the digestion enzyme. False discovery rates at the precursor, peptide, and protein levels were controlled at 1%. Protein abundances were estimated using the MaxLFQ algorithm implemented in Spectronaut.

### Enzyme supplementation by dKO sup

*M6PR*/*IGF2R* double-knockout cells were cultured in normal growth medium in a 10 cm dish until they reached 100% confluency. The medium was replaced with 10 ml of fresh complete DMEM, and cells were cultured for an additional 2 days. Conditioned medium was collected, centrifuged at 2000 rpm for 5 min, and filtered through a 0.22 μm pore filter (180E03; Sartorius). Supernatants were stored at −20 °C until use (dKO sup). For the dKO sup treatment, the conditioned medium was mixed at a 1:1 ratio with complete DMEM and applied to cells for 3 days.

### Western blotting

Cells were lysed in SDS-PAGE sample buffer (56 mM Tris-HCl, pH 6.8, 6% glycerol, 2% SDS, 0.1 M DTT, 0.04% Bromophenol Blue) and boiled for 5 min. Culture supernatants were cleared by centrifugation (2000 rpm, 5 min), mixed with one-third volume of 4x SDS-PAGE sample buffer, and boiled for 5 min. Samples were resolved by SDS-PAGE and transferred to polyvinylidene difluoride membranes (IPVH00010; Merck). Membranes were blocked in Tris-buffered saline containing 0.1% Tween-20 and 3% skim milk and incubated with primary antibodies, followed by horseradish peroxidase-conjugated secondary antibodies. Chemiluminescent signals were developed with Immobilon Forte (WBLUF0100; Merck) and imaged on the ChemiDoc Touch Imaging System (Bio-Rad).

### LC3 flux assay

The LC3 flux assay was conducted according to the autophagy assay guidelines (62). Briefly, confluent cells were washed with PBS and incubated for 2 h in EBSS, either in the presence or absence of 125 nM bafilomycin A1 (BafA1). The cells were then washed with PBS, lysed with SDS-PAGE sample buffer, and subjected to Western blotting. The samples were separated on a 13% acrylamide gel and transferred to a polyvinylidene difluoride membrane. The membranes were incubated separately with antibodies against LC3 and β-actin for detection. Lipidated LC3-II is recruited to the autophagosome membrane upon autophagy activation and is degraded by autophagy. Therefore, the difference in the amount of LC3-II between samples treated with and without BafA1 represents the autophagic degradation of LC3 flux over 2 h.

### Enzymatic deglycosylation analysis

Cell lysate and culture supernatant samples prepared in SDS-PAGE sample buffer were treated with Endo H (P0702S; NEB) or PNGase F (P0704S; NEB) according to the manufacturer’s instructions. Briefly, 10 μl of SDS-PAGE sample was mixed with 1 μl of Endo H or 1 μl of PNGase F and incubated at 37 °C for 1 h. For PNGase F treatment, samples were preincubated in the Glycoprotein denaturing buffer (supplied with the enzyme) at 100 °C for 10 s, and 1% NP-40 was added to the reaction.

### Imaging analysis

Bright-field images were acquired on an IX83 inverted microscope (OLYMPUS) equipped with a UPlanFLN 4 × /0.13 PhL objective and a DP80 digital camera (OLYMPUS). Live cells were imaged directly in culture medium without fixation. For lysosome analysis, cells grown on coverslips for 24 h were treated with 200 nM LysoTracker Red DND-99 (L7528; Invitrogen) for 30 min, then washed with PBS and fixed with 4% PFA for 10 min at room temperature. Cellular cholesterol was visualized by Filipin staining. Cells on coverslips were washed with PBS and fixed with 4% PFA for 10 min at room temperature. Fixed cells were treated with 100 μg/ml Filipin III (F4767; Merck) at room temperature for 1 h. *In situ* β-glucosidase activity was visualized with SPiDER-β-Gal (Dojindo) (63). 1 μM of the SPiDER probe was added to the culture medium for the last 1 h of incubation, and the cells were co-stained with LysoTracker. The coverslips were washed and fixed as described above. After washing with PBS, samples were mounted and imaged either on an IX83 inverted microscope (OLYMPUS) equipped with a PlanApo N 60 × /1.42 NA oil-immersion objective, Zyla sCMOS camera (ANDOR), and U-HGLGPS fluorescence light source (OLYMPUS) or on LSM900 with a PlanApo 63 × /1.4 NA oil-immersion objective lens (Carl Zeiss). Images were acquired using cellSens software (OLYMPUS) or ZEN software (Zeiss), and quantified using Fiji (https://imagej.net/Fiji), or CellProfiler ver. 4.2.8 (https://cellprofiler.org).

### Electron microscopy

Cells were rinsed with phosphate buffer and fixed with 2.5% glutaraldehyde in phosphate buffer for 2 h, followed by post-fixation with 1% osmium tetroxide for 1 h at 4 °C. Samples were dehydrated through a graded ethanol series and embedded in Epon resin for 3 days at 60 °C. Ultrathin sections of 70 nm were cut using an ultramicrotome (EM UC7; Leica) and mounted on mesh grids. Sections were stained with 2% uranyl acetate for 20 min at room temperature, followed by lead citrate staining for 5 min at room temperature. After drying, samples were examined using a transmission electron microscope (JEM-1400, JEOL) at an accelerating voltage of 80 kV.

### Cholesterol quantification

Cellular cholesterol levels were determined using the Amplex Red Cholesterol Assay Kit (A12216; Thermo Fisher Scientific), according to the manufacturer’s instructions with minor modifications. To minimize intracellular cholesterol accumulation derived from the culture medium, cells were pre-cultured in medium supplemented with 2% FBS for 48 h, and then treated with the dKO sup in 5% FBS medium. Cells were lysed in distilled water containing a protease inhibitor cocktail (04693132001; Roche) by sonication. Ten micrograms of each lysate were incubated with the Amplex Red working solution. Fluorescence was measured at Ex/Em 540/580 nm using a microplate reader (Infinite 200 PRO).

### Endosome/lysosome rupture assay

The assay was performed as previously described (64, 65). Effectene (301425, QIAGEN)-coated polystyrene beads (09850; Polysciences, PA) were prepared according to the manufacturer’s instructions, as for DNA transfection. Cells were allowed to internalize the beads for 3 h, then fixed and stained with anti–Gal3 antibody after 3 h and 12 h chase periods. The proportion of damaged endosomes/lysosomes was quantified by counting the total number of internalized beads per cell and determining the fraction that was Gal3.

### Statistics

Parametric comparisons between two groups were conducted using Student’s *t* test or Welch’s *t* test depending on variance. For comparisons among multiple independent groups, parametric data were analyzed using one-way ANOVA, followed by Tukey’s multiple-comparisons test. Nonparametric data were analyzed using the Kruskal-Wallis test, followed by pairwise Mann–Whitney U tests with *p*-values adjusted using the Holm method. A *p*-value of less than 0.05 was considered statistically significant (∗*p* < 0.05, ∗∗*p* < 0.01, ∗∗∗*p* < 0.001). Statistical details are provided in the figure legends, and reported values are presented as means and SD.

## Data availability

The data supporting the findings of this study are available within the article and its supporting information or from the corresponding author upon reasonable request.

## Supporting information

This article contains supporting information.

## Conflict of interest

The authors declare that they have no conflicts of interest with the contents of this article.
