# Supplementary material for: M6PR/IGF2R double-knockout supplies therapeutic sources for mucolipidosis II and Niemann-Pick C2
Source: J Biol Chem. 2026 Aug 31;302(10):113511. doi: 10.1016/j.jbc.2026.113511 (PMC13631582; doi:10.1016/j.jbc.2026.113511)
Supplement: Supplementary file 1 [file mmc2.pdf]

Supporting Information

M6PR/IGF2R double-knockout supplies therapeutic sources for mucolipidosis II and Niemann-Pick C2

Toshiharu Fujita, Seigo Terawaki, Takahito Moriwaki, and Takanobu Otomo

Table of contents

- Figure S1
- Figure S2
- Table S1

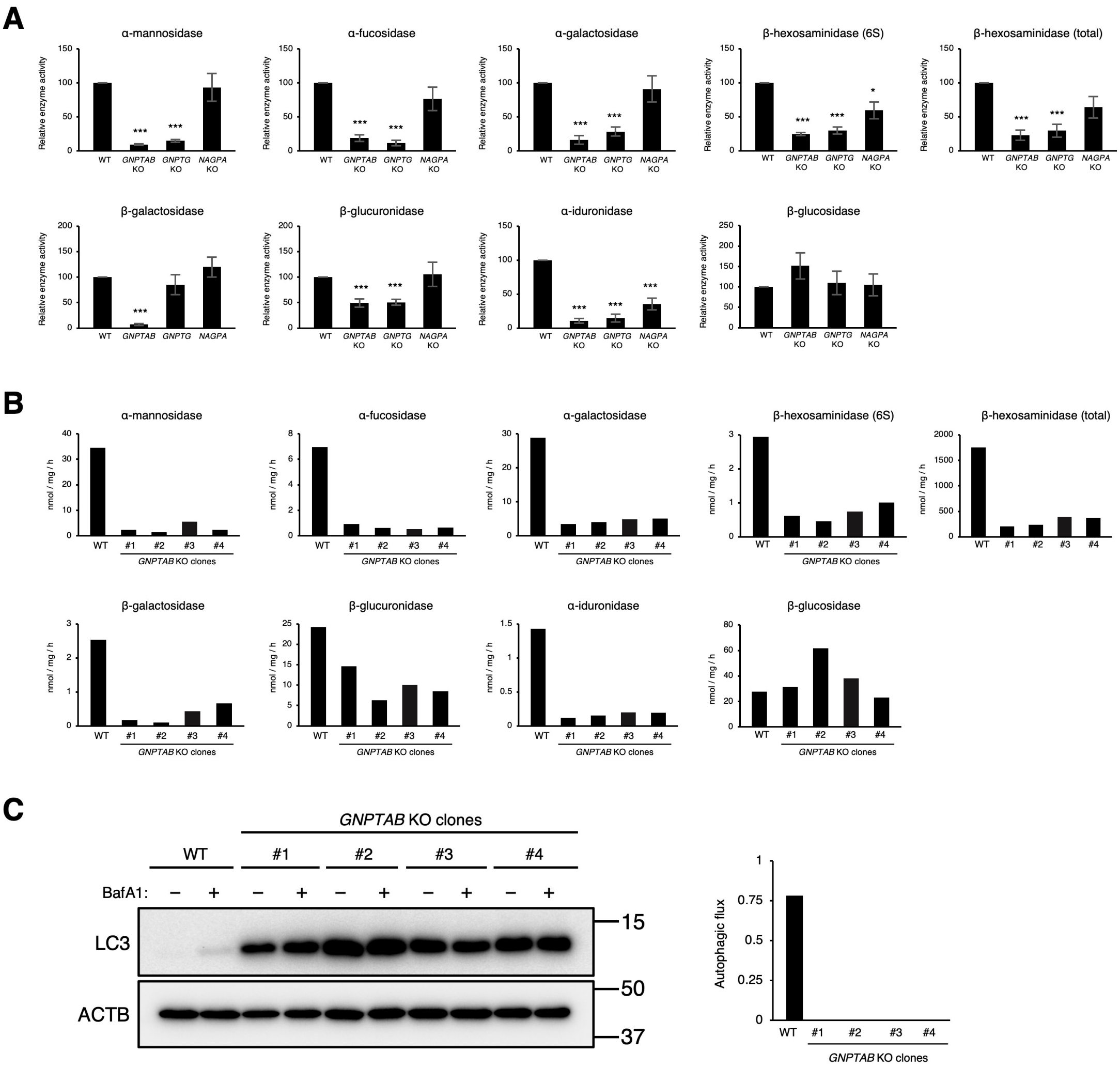

Figure S1. Clonal variation of *GNPTAB* KO HeLa cells as mucolipidosis II models.

(A) Lysosomal enzyme activities of *GNPTAB*, *GNPTG*, and *NAGPA* KO cells. The relative activities of each enzyme to WT are shown in separate bar graphs (mean ± SD, n = 3). Significance was determined between WT and each experimental group with Student's *t*-test (* $p < 0.05$, *** $p < 0.005$). **(B)** Lysosomal enzyme activities in WT and *GNPTAB* KO clones (#1 - #4). **(C)** Autophagic flux of *GNPTAB* KO (#1 - #4) clones. Representative immunoblots of LC3 and β-actin (ACTB) are shown (left). Autophagic flux was calculated from the difference in LC3 protein levels between lysosomal inhibitor BafA1-treated and untreated groups (right). BafA1, bafilomycin A1.

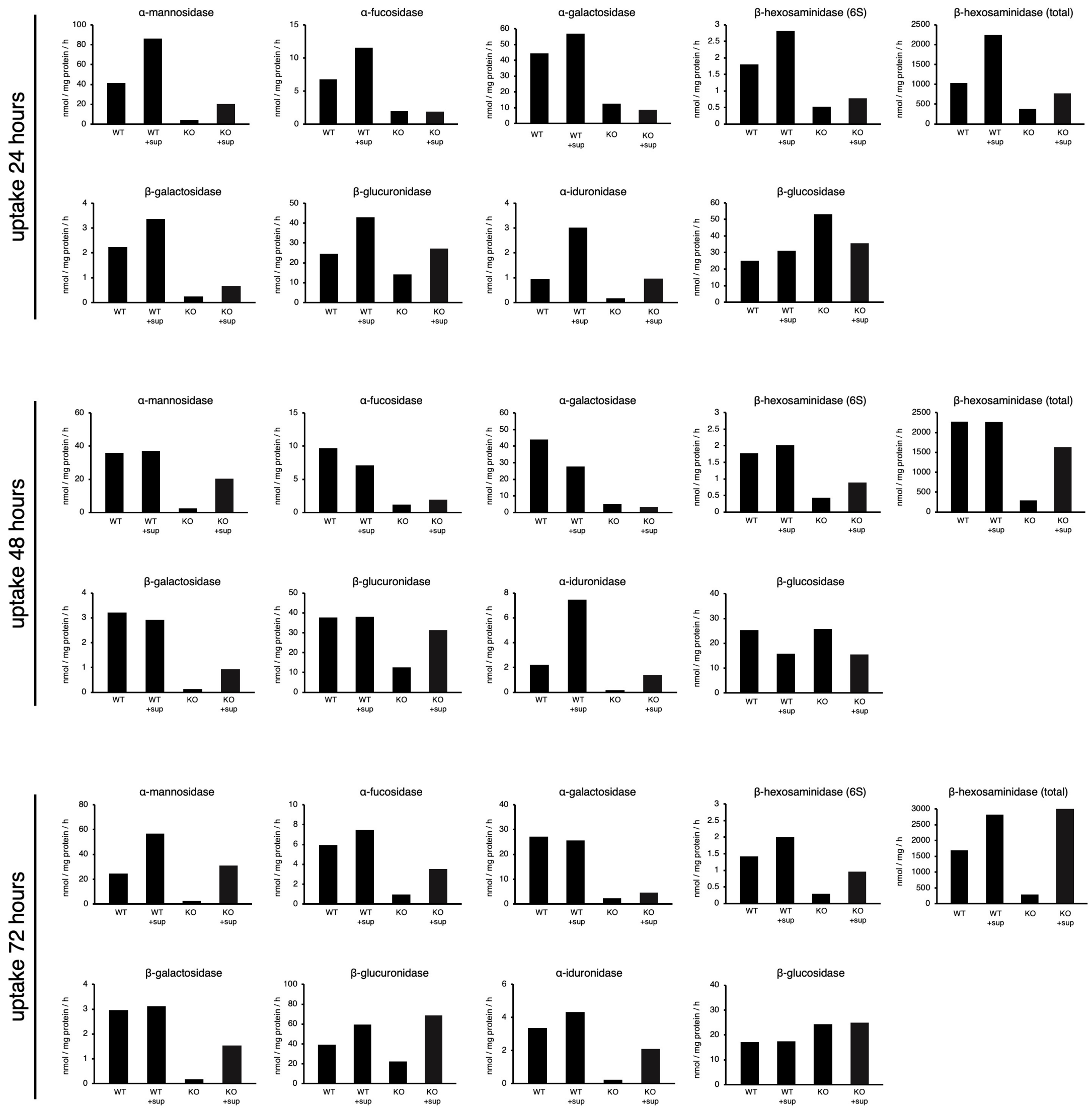

Figure S2. Time course of lysosomal enzyme uptake supplied by dKO sup.

WT and *GNPTAB* KO HeLa cells were treated with dKO sup for the indicated durations, and lysosomal enzyme activities were measured using the 4MU assay at each time point of 24, 48, and 72 h.

Gene name		Mutations	
		allele 1	allele 2
GNPTAB	#1	del 2 bp	del 8 bp
	#2	del 1 bp	homo
	#3	del 1 bp	del 12 bp + ins 3 bp
	#4	del 1 bp	del 15 bp
GNPTG		del 1 bp	homo
NAGPA		del 2 bp	del 11 bp
M6PR		del 2 bp	homo
IGF2R		del 1 bp	homo
M6PR<br>***IGF2R***	dKO	del 2 bp<br>del 7 bp	homo<br>homo
MAN2B1		ins 2 bp	del 5 bp
FUCA1		del 2 bp	del 8 bp
GLB1		ins 2 bp	del 19 bp
IDUA		ins 1 bp	homo
HEXA		del 2 bp	del 5 bp
HEXB		del 1 bp	homo
LIPA		del 1 bp	homo
NPC1		ins 1 bp	del 8 bp
NPC2		ins 1 bp	del 1 bp

Table S1. Genotypes of KO cell clones employed in this study.

The knockout cell lines were established by the CRISPR/Cas9 technique. Target genes are shown with the mutations introduced by gene targeting.
